# The Expression of HIF-1α and VEGF in Radicular Cysts and Periapical Granulomas

**DOI:** 10.1055/s-0044-1795078

**Published:** 2024-12-10

**Authors:** Mohammed Amjed Alsaegh, Shishir Ram Shetty, Okba Mahmoud, Sudhir Rama Varma, Alaa Muayad Altaie, Surendra Singh Rawat

**Affiliations:** 1 Department of Oral and Craniofacial Health Sciences, College of Dental Medicine, University of Sharjah, Sharjah, United Arab Emirates; 2 Research Institute for Medical and Health Sciences, University of Sharjah, Sharjah, United Arab Emirates; 3 Department of Clinical Sciences, College of Dentistry, Ajman University, Ajman, United Arab Emirates; 4 Center for Medical and Bio-allied Health Sciences Research, Ajman University, Ajman, United Arab Emirates; 5 Center of Excellence for Precision Medicine, Research Institute for Medical and Health Sciences, University of Sharjah, Sharjah, United Arab Emirates; 6 College of Medicine, Research and Graduate Studies, Mohammed Bin Rashid University of Medicine and Health Sciences, Dubai, United Arab Emirates

**Keywords:** HIF-1α, VEGF, hypoxia, radicular cyst, periapical granuloma

## Abstract

**Objectives** This study aimed to evaluate the expression levels of hypoxia-inducible factor 1-α (HIF-1α) and vascular endothelial growth factor (VEGF) in radicular cysts and periapical granulomas, thereby contributing to the understanding of their potential significance in the differential diagnosis and treatment of these lesions.

**Materials and Methods** In the present cross-sectional study, 51 samples of periapical lesions were included. Of them, 24 samples were radicular cysts, and 27 samples were periapical granulomas. Samples were immunohistochemically analyzed for HIF-1α and VEGF proteins expression. Chi-square tests and Spearman's rank correlation coefficient tests were used to detect differences and correlations among the parameters, respectively.

**Results** In radicular cysts, HIF-1α expression was absent in 1 (4.2%), weak in 5 (20.8%), mild in 7 (29.2%), and strong in 11 (45.8%) cases, while VEGF expression was absent in 1 (4.2%), weak in 6 (25.0%), mild in 9 (37.5%), and strong in 8 (33.3%) of the cases; nevertheless, in periapical granulomas, HIF-1α expression was absent in 8 (29.6%), weak in 6 (22.2%), mild in 9 (33.3%), and strong in 4 (14.8%) of the cases, whereas VEGF expression was absent in 4 (14.8%), weak in 16 (59.3%), mild in 4 (14.8%), and strong in 3 (11.1%) of the cases. Chi-square test revealed a significant difference in the expression of HIF-1α and VEGF between radicular cysts and periapical granuloma (chi-square test = 8.906, *p* = 0.031; chi-square test = 10.401, *p* = 0.015, respectively). Spearman's correlation test showed a significant correlation between HIF-1α and VEGF in the total samples of both radicular cysts and periapical granulomas (rho = 0.385, *p* = 0.005).

**Conclusion** There is high expression of both HIF-1α and VEGF throughout the odontogenic epithelium and connective tissue of the radicular cyst and periapical granuloma. Both HIF-1α and VEGF are more highly expressed in radicular cysts than in periapical granulomas. These findings may aid in the diagnosis and management of suspected periapical lesions, suggesting that radicular cysts exhibit more advanced hypoxic conditions and associated pathways compared with periapical granulomas.

## Introduction

Among the most common oral pathological lesions in humans are radicular cysts and periapical granulomas. These lesions result in the loss of alveolar bone and the development of granulation tissue in the area surrounding the root's apex. 1 When a root canal infection is persistent, pathogenic bacteria and bioproducts are released into the periradicular tissues. Consequently, periapical granulomas and radicular cysts were developed. Infected root canals, primarily caused by dental caries, lead to continuous antigenic stimulation, which initiates cytokine release and triggers inflammation. Periapical lesions are characterized by inflammation and the infiltration of lymphocytes, macrophages, and plasma cells, representing the body's defense response against microbial invasion. 2 Periapical granulomas are granulation tissue that is sometimes epithelized and infiltrated by lymphocytes, plasma cells, and macrophages. 1 In radicular cysts, however, odontogenic epithelial remnants proliferate forming the epithelial lining. 3 A key predictor of the presence of a periapical cyst, as identified on a dental radiograph, was the involvement of multiple teeth in the periapical lesion. 4

In recent years, scientists have been increasingly interested in understanding the biology and prognosis of periapical lesions. Under a biological scope, cytokines have been associated with characteristics of odontogenic lesions.

As a heterodimer, hypoxia-inducible factor (HIF-1) consists of α and β subunits. HIF-1β is found in the nucleus and is constitutively expressed. 5 On the other hand, HIF-1α is found in the cytoplasm and is a transcription factor that regulates the cellular response to hypoxia. In normal oxygen conditions, it is posttranslationally hydroxylated by enzymes containing the prolyl hydroxylase domain, and then degraded by proteasomes. However, hypoxia interferes with this enzymatic degradation of HIF-1α, causing it to stabilize, translocate to the nucleus, and heterodimerize with HIF-1β. Once the heterodimer binds to specific DNA sequences, the HIF-1 pathway is activated. 6 7 Thus, cellular adaptation and resistance to hypoxia are increased by upregulating multiple target genes. It has been found that HIF-1α plays a key role in the regulation of more than 40 genes in response to hypoxia. Several previous studies have investigated the expression of HIF-1α in radicular cysts and periapical granulomas, 2 8 9 and demonstrate their hypoxic nature. 2 9

One of the major well-studied targets of HIF-1 is the vascular endothelial growth factor (VEGF) pathway. VEGF is involved in a variety of processes, including angiogenic, mitogenic, and permeability-modulating properties. Both endothelial and nonendothelial cells possess VEGF receptors, which act via autocrine pathways to regulate cell survival and function. 10 In addition, VEGF plays an important role in osteoblast differentiation as well as osteoclast function, thereby maintaining bone homeostasis. 11 Furthermore, VEGF induces an increase in microvascular permeability, which is accompanied by plasma protein extravasation and predictable changes in the stroma that promote angiogenesis. 12 There is evidence that VEGF levels are higher in radicular cysts and granulomas than in healthy tissues. 13 14

A clear correlation exists between HIF-1α and VEGF in several physiological 15 and pathological conditions. 16 17 However, periapical lesions did not devote much attention to the significance of this correlation. This study aimed to evaluate the expression levels of HIF-1α and VEGF in radicular cysts and periapical granulomas, thereby contributing to the understanding of their potential significance in the differential diagnosis and treatment of these lesions.

## Materials and Methods

### Study Design, Sample Size, Tissue Samples, and Ethical Approval

The present cross-sectional study included 51 prediagnosed periapical lesion samples, of which 24 were radicular cysts and 27 were periapical granulomas. Samples were collected from the Oral Surgery Clinic at the University of Science and Technology of Fujairah. Baseline data were collected from patients' medical records, including age, gender, lesion location, and the affected tooth or teeth. Lesion size was determined by measuring the maximum craniocaudal and mesiodistal diameters on standard panoramic images, and the arithmetic mean of these measurements was calculated to determine the final cyst size. Diagnoses of inflamed periapical lesions followed the World Health Organization's criteria, incorporating histopathology, clinical history, and radiographic features. Radicular cysts were identified by their location in the periapical area of a tooth with necrotic pulp, without periodontal communication, and a cystic cavity surrounded by nonkeratinizing epithelium with inflammatory cells in the connective tissue. Periapical granulomas were similarly located in the periapical region of a tooth with necrotic pulp, but lacked epithelial lining, instead showing an infiltration of inflammatory cells such as macrophages, lymphocytes, and plasma cells.

In accordance with the Declaration of Helsinki, this study was approved by the ethical approval committee of Ajman University (reference number: H-17-05-08). Consent was obtained from all subjects, with parental consent provided for the 15-year-old patient.

This sample size was calculated through the use of the statistical software G*Power (version 3.1.9.7 software; University of Düsseldorf, Germany). Based on the information provided by da Costa et al, 8 which determined that 15 patients were needed for each group, we opted for a more convenient approach by using an effect size of 0.4, with 80% power and a 5% error rate. Consequently, a total of 51 patients were selected as a convenience sample for this study, with 24 samples from radicular cysts and 27 from periapical granulomas.

### Inclusion and Exclusion Criteria

Patients over the age of 15 years with necrotic pulp and periapical radiolucency of at least 2 mm in either jaw, as well as histopathological confirmation of radicular cysts and periapical granulomas, were included in this study. Pregnant or lactating women, individuals with systemic medical conditions, and those taking medications were excluded from the study. Furthermore, samples were not included if there was a history of acute local infection or if the diagnosis was not confirmed by histopathological analysis.

### Sampling Procedure

All patients were informed of the procedures to be performed prior to the operation and signed informed consent forms. Samples were obtained either through surgical enucleation or following nonrestorable tooth extraction, depending on the clinical circumstances. Smaller, early-stage lesions were typically collected during tooth extraction. Lesions harvested from extracted teeth were carefully isolated using a sterile no. 15 surgical scalpel blade and rinsed in sterile normal saline. In cases where samples were collected via enucleation, the wound site was sutured after bleeding was controlled.

### Immunohistochemistry: Anti-HIF-1α and Anti-VEGFA Antibody Staining

Tissues were fixed in 10% neutral buffered formalin for 8 hours at room temperature and embedded in paraffin. Formalin-fixed paraffin-embedded sections were cut into 4-µm sections and were adhered to positively charged slides (MIC3040, Thermo-Fisher Scientific) by incubation at 37°C overnight. Following deparaffinization, the sections were stained immunohistochemically for HIF-1α and anti-VEGFA antibody expression. The immunohistochemical (IHC) detection of the protein targets was performed using primary antibodies: anti-VEGFA antibody (Abcam cat#AB185238, rabbit monoclonal, clone EP1176Y, 1:200) and anti-HIF-1α antibody (Abcam AB51608, rabbit monoclonal, clone EP1215Y, 1:100). Antigen retrieval was done in pH 9.0 Tris-EDTA solution in a microwave oven at 95°C for 30 minutes. The primed proteins were chromogenically detected with AB64264, Abcam's HRP/DAB (ABC) IHC detection kit. Specimens with known positive antigenicity were used as positive control specimens. For the negative controls, sections of periapical lesions were incubated in normal rabbit serum without the addition of the primary antibody.

Images of the stained sections were acquired with Olympus BX63 microscope equipped with Olympus DP75 camera (resolution of 5,760 × 3,600 pixels and pixel size of 5.86 × 5.86 µm) with Cell Sens Entry software (version 1.17). Images were acquired with ×20 (numerical aperture 0.45; 1,920 × 1,200 pixels; 465.079 nm/pixel resolution in both *x* - and *y* -axes) and ×40 (numerical aperture 0.6; 1,920 × 1,200 pixels; 232.54 nm/pixel resolution in both *x* - and *y* -axes) objective lenses. The IHC staining of HIF-1α and VEGF was yellowish to brown. The IHC presence of HIF-1α and VEGF was assessed within the connective tissue of radicular cysts and periapical granulomas, as well as in the epithelial lining of radicular cysts and, where applicable, in the epithelium of periapical granulomas. Under a light microscope, semiquantitative scoring was conducted. HIF-1α and VEGF immunostaining was assessed by calculating the percentage of positive cells out of the total cells in 10 representative high-power fields. There were four criteria for use: 0 when there was no staining or if it was questionable; weak for results with a positivity of ≥25%, mild for results with a positivity of 26 to 50%, and strong for results with a positivity of more than 50%.

### Statistical Analysis

The Statistical Package for the Social Sciences (SPSS) 28.0 software (IBM SPSS, Armonk, New York, United States) was used to analyze the data. The chi-square test was used to determine whether significant differences existed. The correlation of expressed markers was further analyzed through Spearman's rank correlation coefficient. Results were considered significant if the *p* -value was less than 0.05.

## Results

A total of 51 samples were included in the study. There were 24 samples of radicular cysts and 27 samples of periapical granulomas. Thirty-seven (72.5%) of these samples were found in males and 14 (27.5%) in females. There were 28 cases located in the maxilla (54.9%), while 23 cases (45.1%) were in the mandible. The mean age was 33.67 years ( Table 1 ).

**Table 1:** Clinical features of the studied cases

Variables	Total	Periapical granulomas	Radicular cysts
**Patients (** ***n*** **)**	51	27 (52.9%)	24 (47.1%)
**Age (y)**	Mean = 33.67 ± 9.873 Range = 15–60	Mean = 33.26 ± 10.044 Range = 15–60	Mean = 34.13 ± 9.870 Range = 20–58
**Gender,** ***n*** **(%)**			
**Male**	37 (72.5%)	17 (63.0%)	20 (83.3%)
**Female**	14 (27.5%)	10 (37.0%)	4 (16.7%)
**Location,** ***n*** **(%)**			
**Maxilla**	28 (54.9%)	19 (70.4%)	9 (37.5%)
**Mandible**	23 (45.1%)	8 (29.6%)	15 (62.5%)
**The most offending tooth**		14 = 3 (11.1%) 15 = 2 (7.4%) 16 = 6 (22.2%) 17 = 2 (7.4%) 18 = 2 (7.4%) 22 = 1 (3.7%) 25 = 1 (3.7%) 26 = 1 (3.7%) 27 = 1 (3.7%) 36 = 3 (11.1%) 37 = 1 (3.7%) 44 = 1 (3.7%) 46 = 1 (3.7%) 47 = 2 (7.4%)	11 = 1 (4.2%) 12 = 1 (4.2%) 15 = 1 (4.2%) 17 = 1 (4.2%) 26 = 1 (4.2%) 2 = 2 (8.4%) 28 = 2 (8.4%) 31 = 1 (4.2%) 34 = 1 (4.2%) 35 = 3 (12.5%) 36 = 1 (4.2%) 37 = 1 (4.2%) 44 = 1 (4.2%) 45 = 1 (4.2%) 46 = 6 (25%)
**Presence of pain**		No = 9 (33.3%)	No = 11 (45.8%)
		Yes = 18 (66.7%)	Yes = 13 (54.2%)
**Size**		Mean = 4.26 mm ± 1.457 Range = 2–7 mm	Mean = 8.83 mm ± 6.638 Range = 2–23 mm

In the studied radicular cyst samples, fibrous connective tissue walls were lined with stratified squamous epithelium or with epithelial hyperplasia. A common finding in the studied radicular cyst samples was the presence of inflammatory cells such as neutrophils, plasma cells, and lymphocytes. In several samples of the periapical granulomas, epithelial strands could be seen throughout sections. All periapical granulomas showed significant infiltration of inflammatory cells.

**Fig. 1:**
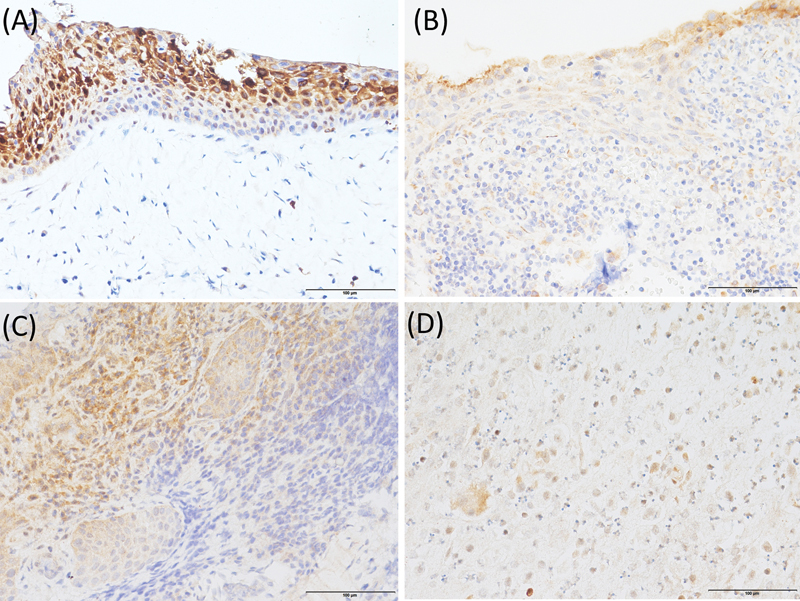
Immunohistochemical staining of HIF-1α in radicular cysts and periapical granulomas (magnification ×400). ( **A** ) Intense HIF-1α nuclear and cytoplasmic staining throughout the whole layers of the odontogenic epithelium of radicular cysts, ( **B** ) less intense HIF-1α cytoplasmic and nuclear staining of epithelium with several immunostained inflammatory infiltrates in the radicular cysts, ( **C** ) intense nuclear and cytoplasmic HIF-1α staining of the positive cells throughout the periapical granulomas, and ( **D** ) less intense HIF-1α staining of the positive cells throughout the periapical granulomas. HIF-1α, hypoxia-inducible factor 1-α.

**Fig. 2:**
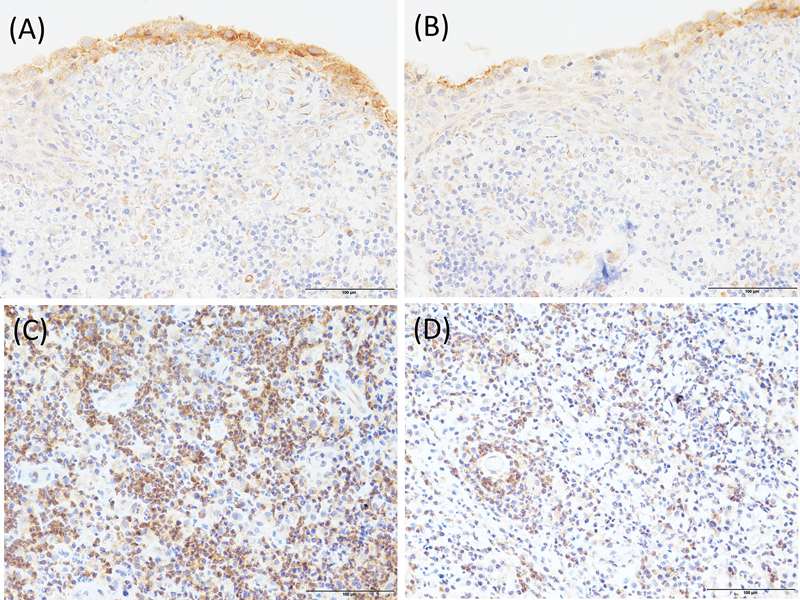
Immunohistochemical staining of VEGF in radicular cysts and periapical granulomas (magnification ×400). ( **A** ) Intense cytoplasmic VEGF staining throughout the layers of the odontogenic epithelium of radicular cysts with few positive inflammatory infiltrates in the connective tissue, ( **B** ) less intense VEGF staining throughout the layers of the odontogenic epithelium of radicular cysts with few positive inflammatory infiltrates in the connective tissue, ( **C** ) intense VEGF staining of the positive cells throughout the periapical granulomas, and ( **D** ) less intense VEGF staining of the positive cells throughout the periapical granulomas. VEGF, vascular endothelial growth factor.

In radicular cysts, HIF-1α was present both in the cytoplasm and nucleus, with greater positivity noted in the nucleus than in the cytoplasm ( Fig. 1 ). Nevertheless, VEGF was found primarily in the cytoplasm ( Fig. 2 ). Expression of both HIF-1α and VEGF was observed in both cystic epithelium and cystic stroma including inflammatory cells. Many of the periapical granulomas examined were positively immunolabeled for HIF-1α both in the cytoplasm and nucleus of the cells, with a higher level of positivity noted in the cytoplasm than in the nucleus ( Fig. 1 ). Meanwhile, VEGF immunopositivity was cytoplasmic in periapical granulomas. Fibroblasts, epithelial, endothelial, and inflammatory cells showed reaction products, although the intensity of immunostaining by the anti-VEGF antibody varied ( Fig. 2 ). The periapical granulomas, however, displayed the highest number of VEGF-positive inflammatory cells compared with the radicular cysts.

In radicular cysts, HIF-1α expression was absent in 1 (4.2%), weak in 5 (20.8%), mild in 7 (29.2%), and strong in 11 (45.8%) cases ( Table 2 ). VEGF expression was absent in 1 (4.2%), weak in 6 (25.0%), mild in 9 (37.5%), and strong in 8 (33.3%) cases ( Table 2 ); nevertheless, in periapical granulomas, HIF-1α expression was absent in 8 (29.6%), weak in 6 (22.2%), mild in 9 (33.3%), and strong in 4 (14.8%) of the cases ( Table 2 ). The expression of VEGF was absent in 4 (14.8%), weak in 16 (59.3%), mild in 4 (14.8%), and strong in 3 (11.1%) cases ( Table 2 ).

**Table 2:** Expression of HIF-1α and VEGF in radicular cysts and periapical granulomas

	Radicular cysts ( *n* = 24)				Periapical granulomas ( *n* = 27)			
	Absent	Weak	Mild	Strong	Absent	Weak	Mild	Strong
HIF-1α	1 (4.2%)	5 (20.8%)	7 (29.2%)	11 (45.8%)	8 (29.6%)	6 (22.2%)	9 (33.3%)	4 (14.8%)
VEGF	1 (4.2%)	6 (25.0%)	9 (37.5%)	8 (33.3%)	4 (14.8%)	16 (59.3%)	4 (14.8%)	3 (11.1%)

Abbreviations: HIF-1α, hypoxia-inducible factor 1-α; VEGF, vascular endothelial growth factor.

The chi-square test revealed a highly significant difference in the expression of HIF-1α and VEGF between radicular cysts and periapical granulomas (chi-square test = 8.906, *p* = 0.031; chi-square test = 10.401, *p* = 0.015, respectively). Spearman's correlation test showed a significant correlation between HIF-1α and VEGF in total samples of radicular cysts and periapical granulomas (rho = 0.385, *p* = 0.005).

## Discussion

Proliferation and cellular aggregates in periapical lesion may limit oxygen diffusion to the center of the island, resulting in hypoxia. This hypoxia affects many cellular processes, including proliferation, angiogenesis, apoptosis, necrosis, and cell survival. 9 VEGF has been identified as a key downstream target of HIF-1α. 7 A complex interaction between cells, cytokines, and other inflammatory factors may contribute to the development of radicular cysts and periapical granulomas.

We observed a high expression of HIF-1α protein in both radicular cysts and periapical granulomas, with significantly greater expression in radicular cysts compared with periapical granulomas. Conversely, a previous study reported comparable expressions of HIF-1α in radicular cysts and periapical granulomas. 2 Meanwhile, previous research has shown that HIF-1α is overexpressed in radicular cysts compared with dental follicles, 8 healthy dental pulp, 2 and healthy gingival tissue. 9 Interestingly, ameloblastomas and odontogenic keratocysts also exhibit higher levels of HIF-1α than dental follicles and gingival tissues. 8 9 18 Moreover, the highest level of expression of HIF-1α was found in radicular cysts compared with ameloblastomas and odontogenic keratocysts. 8 This variation in findings from previous studies suggests inconsistency in HIF-1α expression across different research and highlights the need for further investigation. It also supports the involvement of hypoxia in the pathogenesis of various odontogenic lesions and implies that radicular cysts may experience a more pronounced hypoxic environment, potentially playing a key role in their development and persistence.

According to previous studies, the expression of HIF-1α in odontogenic cysts and periapical granulomas could be viewed as a cause of the pathology or a response to it. In fact, increased hypoxia occurring in odontogenic lesions was attributed to ischemia during the development of radicular cysts and periapical granulomas. 2 9 Interestingly, HIF-1α and caspase-3 immunoexpression and immunolocalization patterns suggest a strong association between hypoxia, apoptosis, and cyst formation. 8 Additionally, phosphatidylinositol 3-kinase/protein kinase B signaling may be stimulated by increased phosphatidylinositol-4,5-bisphosphate 3-kinase catalytic subunit α and decreased phosphatase and tensin homolog expression, which promote cell survival and support the mechanism of cyst formation under hypoxia conditions. 9 On the other hand, apoptosis is an adaptive cellular response to cellular stress, such as hypoxia, that permits organisms to eliminate unwanted or dysfunctional cells. 19 Similarly, hypoxia-related proteins may induce autophagy in periapical lesions, which may have a direct role in the development and maintenance of periapical lesions. 2 It is noteworthy that Szaraz et al proposed that odontogenic cysts may be influenced by molecular mechanisms associated with primary cilia and hypoxia in similar ways to autosomal dominant polycystic kidney disease. 20

HIF-1α activation is thought to contribute to host defense against periapical lesions by downregulating bone resorption cytokines, M1 macrophages, and nuclear factor-κB, which is associated with osteoclastogenesis. 1 Furthermore, the HIF-1 pathway plays an essential role in neoangiogenesis and bone healing. 21 Meanwhile, it has been proposed that hypoxia does not initiate cellular proliferation, but rather involves an adaptive pathway, which is necessary for future proliferation. 19

The present study found that HIF-1α is expressed in both the cytoplasm and nucleus of immunopositive radicular cyst cells, with the nucleus being the most abundant. In contrast, periapical granulomas also express HIF-1α in the cytoplasm and nucleus, although its expression is more pronounced in the cytoplasm. Consistent with our results, a prior investigation revealed the presence of HIF-1α in both the cell nucleus and cytoplasm of immunopositive cells within radicular cysts. 8 Indeed, under normoxic conditions, HIF-1α is primarily expressed within the cytoplasm and undergoes rapid degradation. However, when hypoxia occurs, it is translocated to the nucleus where it is activated. Hence, HIF-1α is theoretically inactive in the cytoplasm. 19 Remarkably, research has revealed the presence of HIF-1α in the cystic regions of ameloblastomas, located in both the nuclei and cytoplasm. Conversely, within solid regions of ameloblastomas, HIF-1α expression was solely localized to the nuclei. This observation indicates that the retention of HIF-1α in the cytoplasm may impede adaptation to hypoxic conditions, potentially triggering apoptosis and the subsequent formation of cystic cavities. 18

The present study findings showed expression of HIF-1α protein in the epithelial cells, inflammatory cells, and fibroblasts of the periapical lesions. Similar findings have been reported in previous studies. 2 8 The increased nuclear localization of HIF-1α expression in radicular cysts may suggest increased HIF-1α activity within these cysts. This potential activity could be reflected in the present study by the elevated expression of VEGF in radicular cysts compared with periapical granulomas.

Previous studies found that the expression of VEGF and its implication in odontogenic cysts was controversial. The expression of VEGF in radicular cysts and dentigerous cysts was comparable. 12 A previous study, however, found that VEGF expression was higher in radicular cysts than dentigerous cysts, possibly because of the inflammatory process involved in radicular cysts. 22 On the other hand, odontogenic keratocysts expressed more VEGF than radicular cysts 12 and dentigerous cysts, 12 23 indicating that angiogenesis may have a significant effect on clinical outcomes of odontogenic keratocysts. 23 Again, contrary results were found in another report that showed greater expression of VEGF in radicular cysts than in odontogenic keratocysts. 24 According to a previous study, radicular cysts and periapical granulomas expressed comparable levels of VEGF. 14 In addition, radicular cysts contained higher levels of VEGF than periapical granulomas, despite the lack of statistical significance. 25

The contradictory findings of VEGF expression in odontogenic cysts and periapical granulomas could be attributed to differences in the study design and methodology used in previous studies, while the testing methodology has been temporarily improved in terms of sensitivity. Additionally, heterogeneity of lesions depending on the type and stage of the lesion, as well as patient characteristics and interpretation biases are also nonneglectable factors. A rigorous research design, standardization of methodology, larger sample sizes, and a comprehensive analysis of patient and lesion characteristics will be necessary to resolve these discrepancies and improve understanding of VEGF expression in odontogenic lesions. Furthermore, meta-analyses and systematic reviews can be used to synthesize existing research and identify potential sources of variation between studies.

The present results indicating a significant correlation between HIF-1α and VEGF in the radicular cysts and periapical granulomas provide novel insights into the pathogenesis of these odontogenic lesions and support the hypothesis that HIF-1α, a marker of hypoxia, may trigger or enhance the production of VEGF. 10 The findings suggest that these lesions may expand and persist under hypoxic conditions, where the activation of HIF-1α leads to the upregulation of VEGF, causing new blood vessels to form to meet the metabolic demands of cystic or granulomatous tissue. The angiogenic response may contribute to the maintenance of the lesion's structure and survival.

The pathogenesis and enlargement of periapical granulomas and radicular cysts are significantly affected by VEGF through several mechanisms. This role of VEGF has been emphasized in several previous studies, including those regarding radicular cysts 14 25 26 27 28 and periapical granulomas. 14 25 29 VEGF in cystic lesions may increase angiogenesis and vascular hyperpermeability, resulting in the accumulation of inflammatory cells and cystic fluid. 24 25 26 30 Eventually, this will lead to increased cystic pressure and the expansion of the cyst. 12 In addition, upregulation of VEGF receptors in endothelial cells occurred during the expanding phase of experimentally induced periapical lesions, suggesting that VEGF is one of the modulators of angiogenesis that plays a role in the development of periapical lesions. 13 It has been little studied how angiogenesis interacts with the skeletal system in cysts. 25 It is possible that the variation in VEGF observed in the fibrous capsules of radicular cysts and dentigerous cysts is a result of different osteolytic activities in these lesions. 22

A majority of the studied radicular cysts and periapical granulomas displayed immunopositive cytoplasmic reactions to VEGF in the present study. Additionally, we found expression of VEGF in both epithelium and connective tissue capsules of radicular cysts and periapical granulomas. Similarly, previous studies have found expression of VEGF in both epithelium and connective tissue of periapical granulomas 14 26 and radicular cysts. 14 25 26 27 28 31 Moreover, stromal cells expressed higher levels of VEGF than epithelial cells. 27

In the present study, there was significant variation in the intensity of immunostaining for VEGF in fibroblasts, epithelial cells, endothelial cells, and inflammatory cells. More inflammatory cells were immunopositive for VEGF in periapical granulomas than in radicular cysts. A previous study found that almost no inflammatory cells were immunolabeled for VEGF in radicular cysts. Additionally, VEGF-positive inflammatory cells were observed in periapical granulomas, but their number decreased with the increase in epithelial components of the lesions. 26 Moreover, the lowest level of VEGF immunoexpression was observed in lesions with few inflammatory infiltrates. 25 In contrast to these findings, a previous study found that inflammation and VEGF expression are negatively correlated in periapical granulomas. 29 In addition, radicular cysts and residual cysts showed strong epithelial expression of VEGF, regardless of the level of inflammatory infiltrate. 28

A limitation of the present study is that it is a cross-sectional observational study. A cause-and-effect model would be more predictably useful in investigating the pathways of HIF-1α in periapical lesions. It would be beneficial to explore additional pathways of hypoxia and HIF-1α in periapical lesions in future studies. The present study focuses exclusively on the histopathological and immunobiological characteristics of the samples. However, it would be ideal if clinical findings and follow-up results were included in the report. Finally, periapical lesions include reactive tissues and inflammatory cysts that replace healthy bone, so there is no true tissue equivalent to serve as a control. 32

In the light of high expression of HIF-1α and VEGF in radicular cysts and periapical granulomas, one could suggest evaluating the nonsurgical treatment of these lesions using anti-HIF-1α and anti-VEGF therapy. Further, intralesional injection of these medications into radicular cysts and periapical granulomas may reduce bone resorption and enhance bone formation in periapical lesions. Edaravone has previously been reported to reduce bone resorption along with decreasing angiogenesis in arthritis. This is believed to be related to the HIF-1α–VEGF–ANG-1 axis. 33 There is also evidence that VEGF might be regarded as a therapeutic target candidate to inhibit bone resorption, angiogenesis, as well as immune responses in periodontitis, periimplantitis, and apical periodontitis. 10 Intriguingly, a previous study found that combining anti-VEGF bevacizumab with moxifloxacin on weekly basis along with standard care resulted in rapid regression of highly expressed VEGF-tubercular granulomas. 34

## Conclusion

There is high expression of both HIF-1α and VEGF throughout the odontogenic epithelium and connective tissue of the radicular cyst and periapical granuloma. Both HIF-1α and VEGF are more highly expressed in radicular cysts than in periapical granulomas. These findings may aid in the diagnosis and management of suspected periapical lesions, suggesting that radicular cysts exhibit more advanced hypoxic conditions and associated pathways compared with periapical granulomas.
